# Proteomic and microRNA Transcriptome Analysis revealed the microRNA-SmyD1 network regulation in Skeletal Muscle Fibers performance of Chinese perch

**DOI:** 10.1038/s41598-017-16718-2

**Published:** 2017-11-28

**Authors:** WuYing Chu, FangLiang Zhang, Rui Song, YuLong Li, Ping Wu, Lin Chen, Jia Cheng, ShaoJun Du, JianShe Zhang

**Affiliations:** 1 Department of Bioengineering and Environmental Science, Changsha University, Changsha, Hunan 410003 China; 2 Collaborative Innovation Center for Efficient and Health Production of Fisheries in Hunan Province, Changde, 415000 China; 3 Institute of Hunan Aquaculture and Fisheries, Changsha, 410005 China; 4 Institute of Marine and Environmental technology, Department of Biochemistry and Molecular Biology, University of Maryland School of Medicine, Baltimore, MD, United States

## Abstract

Fish myotomes are comprised of anatomically segregated fast and slow muscle fibers that possess different metabolic and contractile properties. Although the expression profile properties in fast and slow muscle fibers had been investigated at the mRNA levels, a comprehensive analysis at proteomic and microRNA transcriptomic levels is limited. In the present study, we first systematically compared the proteomic and microRNA transcriptome of the slow and fast muscles of Chinese perch (*Siniperca chuatsi*). Total of 2102 proteins were identified in muscle tissues. Among them, 99 proteins were differentially up-regulated and 400 were down-regulated in the fast muscle compared with slow muscle. MiRNA microarrays revealed that 199 miRNAs identified in the two types of muscle fibers. Compared with the fast muscle, the 32 miRNAs was up-regulated and 27 down-regulated in the slow muscle. Specifically, expression of miR-103 and miR-144 was negatively correlated with SmyD1a and SmyD1b expression in fast and slow muscles, respectively. The luciferase reporter assay further verified that the miR-103 and miR-144 directly regulated the SmyD1a and SmyD1b expression by targeting their 3′-UTR. The constructed miRNA-SmyD1 interaction network might play an important role in controlling the development and performance of different muscle fiber types in Chinese perch.

## Introduction

The fast-twitch and slow-twitch muscle fibers are the two major types of fish skeletal muscle^1^. The fast contracting fibers are deep, anaerobic and white muscle that permits sudden bursts of motion, whereas the slow-contracting fibers are superficial, aerobic and red muscle that permits sustained locomotion over long periods^2^. Differences between the fast and slow muscle fibers in functionality and physiology are well documented^3,4^, but the molecular regulation of their maintenance in adult fish remains unclear.

MicroRNAs (miRNAs) are groups of small, single-stranded, non-coding RNAs. Their major function involves mediating the posttranscriptional silencing of target genes^5^. The highly muscle-enriched or muscle-specific microRNAs (miRNAs) are referred to as myomiRs, such as miR-1, miR-133a, miR-133b, miR-206, miR-208, miR-208b, miR-486 and miR-499^6–11^. Recent studies demonstrated that myomiRs may play an important role in the regulation of muscle fiber type specification and maintenance in some vertebrate species^12,13^. The distinct characteristics of the myosin heavy chain (MyHC) isoforms are necessary for defining specific types of muscle fiber^14^. MiR-499 was identified to regulate the expression of the slow MyHC, thus governing the slow muscle fiber type phenotype^9,10^. MiR-214 in zebrafish was reported to regulate the slow muscle phenotype by targeting suppressor of fused (Sufu), a negative regulator of hedgehog signaling^15^. Our recent findings revealed that miR-143 silencing leads to the up-regulation of MyoD and fast MyHC gene expression in Chinese perch. MyoD is a member of the myogenic regulatory factors (MRFs), which exerts a central role in the determination, terminal differentiation and lineage maintenance of vertebrate skeletal muscle^16^. Therefore, identifying miRNAs and their target genes that control muscle development is essential for better understanding the regulatory mechanism of miRNA function in muscle fiber specification and maintenance.

It is known that histone modification plays important roles in transcriptional regulation. Recent studies revealed that SET domain-containing proteins could modulate transcription by methylating unacetylated lysine residues on histone tails^17^. It has been shown that SmyD1, a histone emthyltransferase is essential for myogenesis in mouse and fish, which have two highly conserved structural and functional domains, namely the SET(Su(var)3–9, enhancer of zeste and trithorax, resulting in translocation) and MYND domains(a cysteine-rich zinc finger motif)^18–21^. The aims of this study were to analyze the proteomic and microRNA transcriptomes of slow and fast muscles of Chinese perch (*Siniperca chuatsi*) and to identify differentially expressed miRNAs and proteins between these two types of muscle fibers, and to further assay how differentially miRNAs target specific muscle functional genes in regulating muscle development.

## Results

### Differential protein expression between the fast and slow muscle fibers

To obtain insights into the molecular differences between slow and fast muscles, we employed an iTRAQ-based quantitative proteomic approach to analyze the proteomic differences in slow and fast muscle fibers. In total, 328763 spectra were generated. Based on the spectral data, 9711 peptides and 2102 proteins were identified with MascotPercolator^22^ (Table 1). Moreover, we found 99 proteins were differentially up-regulated and 400 were down-regulated (Fold change ≥ 1.2 or ≤0.83) in fast muscle compared with slow muscle by iTRAQ (Supplementary Table S1). Among these differentially expressed proteins, twenty four proteins have been implicated in controlling the performance of the two muscle fiber types (Table 2).

**Table 1:** The whole protein identified by iTRAQ.

Observation	Number	Fold change	Q-value
Total spectra	328763		≤0.01
Spectra	50620		≤0.01
Unique spetra	36864		≤0.01
Peptide	9711		≤0.01
Unique peptide	8403		≤0.01
Protein identified	2102		≤0.01
Significant regulated	499	≥1.2 or ≤0.83	≤0.05
Up-regulated(FM/SM)	99	≥1.2	≤0.05
Down-regulated(FM/SM)	400	≤0.83	≤0.05

Proteins with 1.2 fold change and Q-value less than 0.05 were determined as differentially expressed protein, and final differentially expressed proteins must be defined in at least 2 replicate expriment. FM/SM means Fast muscle *vs* Slow muscle.

**Table 2:** The 24 differentially expressed proteins associated with the performance of different muscle fiber types.

Name	Ratio	Regulate (FM/SM)	Protein Coverage(%)	NCBInr Accession	Description
Actinin alpha 1	1.71	Up	99.9	gi|67462109|sp|P68140.1|ACTSA_TAKRU	F-actin cross-linking protein
Actinin alpha 3	1.34	Up	92.4	gi|348520157|ref|XP_003447595.1|	F-actin cross-linking protein
Myomesin 1	1.99	Up	53.7	gi|348500625|ref|XP_003437873.1|	Sarcomeric M-band protein
Myomesin 2	1.93	Up	61.2	gi|432843412|ref|XP_004065623.1|	Sarcomeric M-band protein
Myosin light chain 1	1.81	Up	99.9	gi|213492442|gb|ACJ47229.1|	Motor contractile protein
Myosin light chain 2	3.3	Up	99.9	gi|213492444|gb|ACJ47230.1|	Motor contractile protein
Myosin-binding protein C	1.71	Up	77.8	gi|317419297|emb|CBN81334.1|	Thick filament-associated protein
SmyD1a	2.21	Up	37.2	gi|348505212|ref|XP_003440155.1|	Methylates histone H3 at Lys-4
Titin	2.5	Up	56.4	gi|348541917|ref|XP_003458433.1|	Giant sarcomeric protein
Tropomodulin 4	2.09	Up	42.6	gi|348525612|ref|XP_003450316.1|	Actin filament assembly
Tropomyosin 2	3.54	Up	99.9	gi|339896195|gb|AEK21799.1|	F-actin cross-linking protein
Actinin alpha 2	0.27	Down	73.7	gi|348501572|ref|XP_003438343.1|	F-actin cross-linking protein
Desmin	0.46	Down	26.1	gi|348518121|ref|XP_003446580.1|	Intermediate filament protein
Kelch repeat and BTB domain-containing protein 5	0.53	Down	10.6	gi|317419950|emb|CBN81986.1|	E3 ubiquitin ligase complex substrate-specific adapter
Myomesin 3	0.33	Down	37.8	gi|348526185|ref|XP_003450601.1|	Sarcomeric M-band protein
Myosin heavy chain 1	0.22	Down	90.9	gi|410902787|ref|XP_003964875.1|	Motor contractile protein
Myosin heavy chain 2	0.13	Down	99.9	gi|211578412|ref|NP_001096096.2|	Motor contractile protein
Myosin heavy chain 3	0.1	Down	81.9	gi|333108579|gb|AEF15872.1|	Motor contractile protein
Myosin heavy chain 4	0.2	Down	99.9	gi|410932121|ref|XP_003979442.1|	Motor contractile protein
Myosin heavy chain 6	0.11	Down	99.9	gi|116062141|dbj|BAF34701.1|	Motor contractile protein
Myosin heavy chain 7	0.13	Down	99.9	gi|239937537|ref|NP_001155228.1|	Motor contractile protein
SmyD1b	0.61	Down	21.4	gi|348517231|ref|XP_003446138.1|	Methylates histone H3 at Lys-4
Tropomodulin 1	0.14	Down	24	gi|410918307|ref|XP_003972627.1|	Actin filament assembly
Tropomyosin 1	0.14	Down	99.9	gi|221219564|gb|ACM08443.1|	Binds to actin filament

FM/SM means Fast muscle *vs* Slow muscle. Compared Fast muscle with Slow muscle, 11 proteins were up-regulated and 13 proteins were down-regulated (Fold change ≥ 1.2 or ≤0.83 with Q-value ≤ 0.05).

### Bioinformatics analysis of differentially expressed proteins

GO analysis was performed to functionally classify the differentially expressed proteinsaccording to various biological process (BP), cellular components (CC), and molecular functions (MF) using the Blast2GO software (v4.5 pipeline 5). The differentially expressed proteins were categorized into 21 subcategories according to their BP. These biological processes included cellular process (20.08%), metabolic process (18.57%), single-organism process (12.38%) and localization (5.23%). According to the cellular components, five main cellular components were identified including cell part (21.11%), cell (21.11%), organelle (16.09%), organelle part (11.94%) and macromolecular complex (10.90%). Based on molecular functions, six key molecular functions were identified including binding (43.47%), catalytic activity (40.27%), transporter activity (8.27%), structural molecular activity (4.00%), electron carrier activity (2.13%) and enzyme regulator activity (1.60%) as shown Supplementary Figure S1.

The differentially expressed proteins in the fast and slow muscles were classified into 178 kinds of KEGG pathways with majority of the proteins associated with metabolic pathways (31.78%), and other seven main pathways were: dilated cardiomyopathy (25.33%), hypertrophic cardiomyopathy (HCM) (25.11%), cardiac muscle contraction (15.11%), Oxidative phosphorylation (12.44%), Regulation of actin cytoskeleton (11.11%), Tight junction (10.22%), Vascular smooth muscle contraction (9.11%) as Supplementary Table S2.

### Differentially expressed miRNAs between the fast and slow muscle fibers

**Figure 1:**
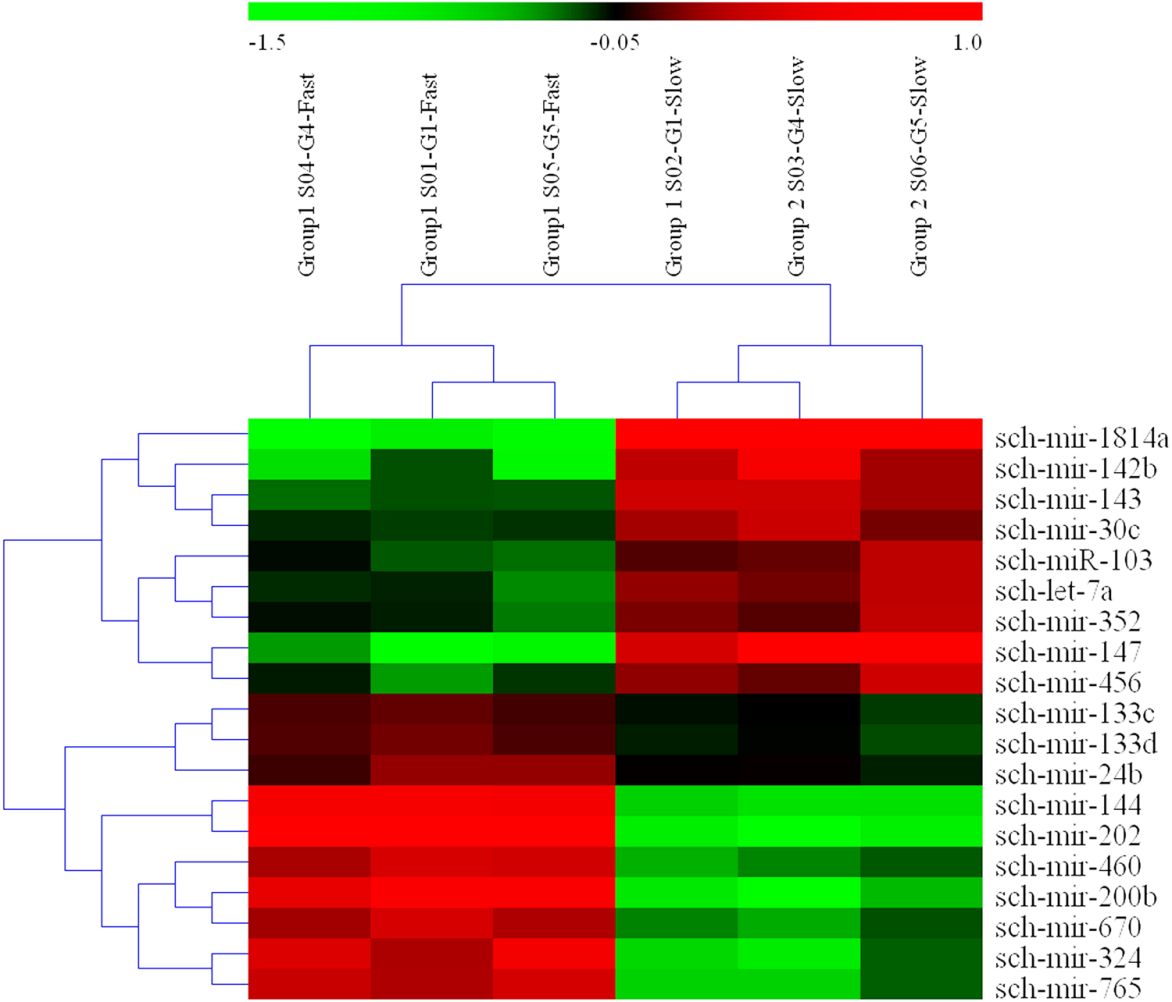
Heat-map of 19 miRNAs differentially expressed in fast muscle and slow muscle based on the microarray analysis (*p* < 0.05). Red indicates that a gene is highly expressed at that stage, whereas green indicates the opposite. The absolute signal intensity ranges from −1 to 1, with corresponding color changes from blue to green, yellow and red. The signal of expression was detected by microarray with three probe repeats.

MiRNA microarrays were employed to characterize the miRNA expression profiles in the white and red muscles and the obtained miRNA data were deposited in GEO dataSets with accession number GSE97173. Among the 199 miRNAs identified in these two types of muscle fibers (Supplementary Table S3), 59 miRNAs showed significant difference in their levels of expression. Compared with the fast muscle, 32 miRNAs were up-regulated and 27 down-regulated. 19 differentially expressed miRNAs reported in controlling the performance of different muscle fiber types were used to construct the heat map (Fig. 1).

### Expression of miR-103 and miR-144 in fast and white muscles with Quantitative real-time PCR

**Figure 2:**
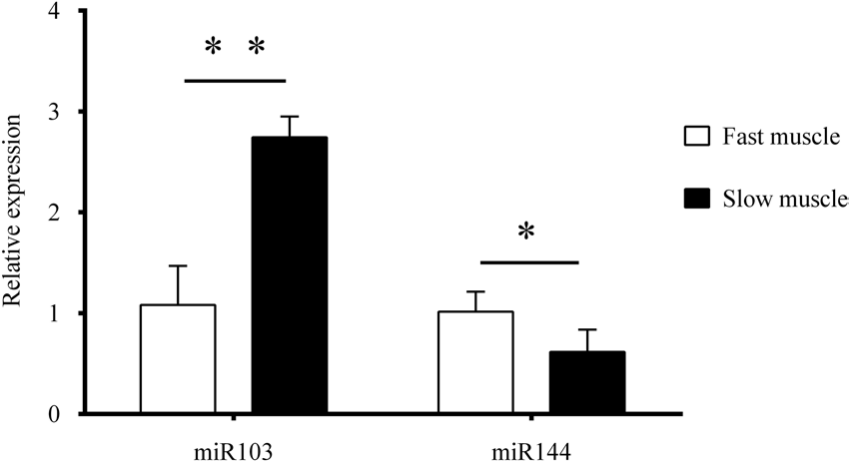
Expression of miR103 and miR144 in fast and slow muscles with Quantitative real-time PCR. Compared with fast muscle (full bars), miR-103 was quite significantly higher expressed in slow muscle (empty bars) (*p* < 0.01), and miR-144 was significantly lower expressed in slow muscle (*p* < 0.05).

SYBR Green qPCR was performed to detect miR-103 and miR-144 levels in the two types of muscle tissues. The results showed that miR-103 expression was significantly higher in slow muscles compared with fast muscles (*p* < 0.05). On other hand, the expression of miR-144 was significantly lower in slow muscles compared with the fast muscles (*p* < 0.05) (Fig. 2).

### Prediction of miRNAs Targeting SmyD1a and SmyD1b

Through partial sequence complementarity, miRNAs regulate gene expression in the posttranscriptional levels by binding to the 3′-UTR of target mRNAs. Based on sequence analysis, the 3′-UTR in the SmyD1a gene contained the evolutionarily conserved binding sites for sch-miR-103, sch-miR-107a, sch-miR-107b, sch-miR-130a, sch-miR-130b, sch-miR-130c, sch-miR-133a-3p, sch-miR-133b-3p, sch-miR-223, sch-miR-7, sch-miR-731, sch-miR-129-5p. The 3′-UTR in the SmyD1b gene contained the evolutionarily conserved binding sites for sch-miR-101a, sch-miR-101b, sch-miR-142a-5p, sch-miR-142b-5p, sch-miR-144, sch-miR-199-3p and sch-miR-338. Interestingly, SymD1a and SmyD1b exhibited an opposite pattern of expression with sch-miR-103 and sch-miR-144 in red and white muscles, respectively, suggesting that SymD1a and SmyD1b expression might be regulated by different microRNAs.

### MiR-103 and miR-144 act directly at the 3′-UTR of the SmyD1a and SmyD1b genes

**Figure 3:**
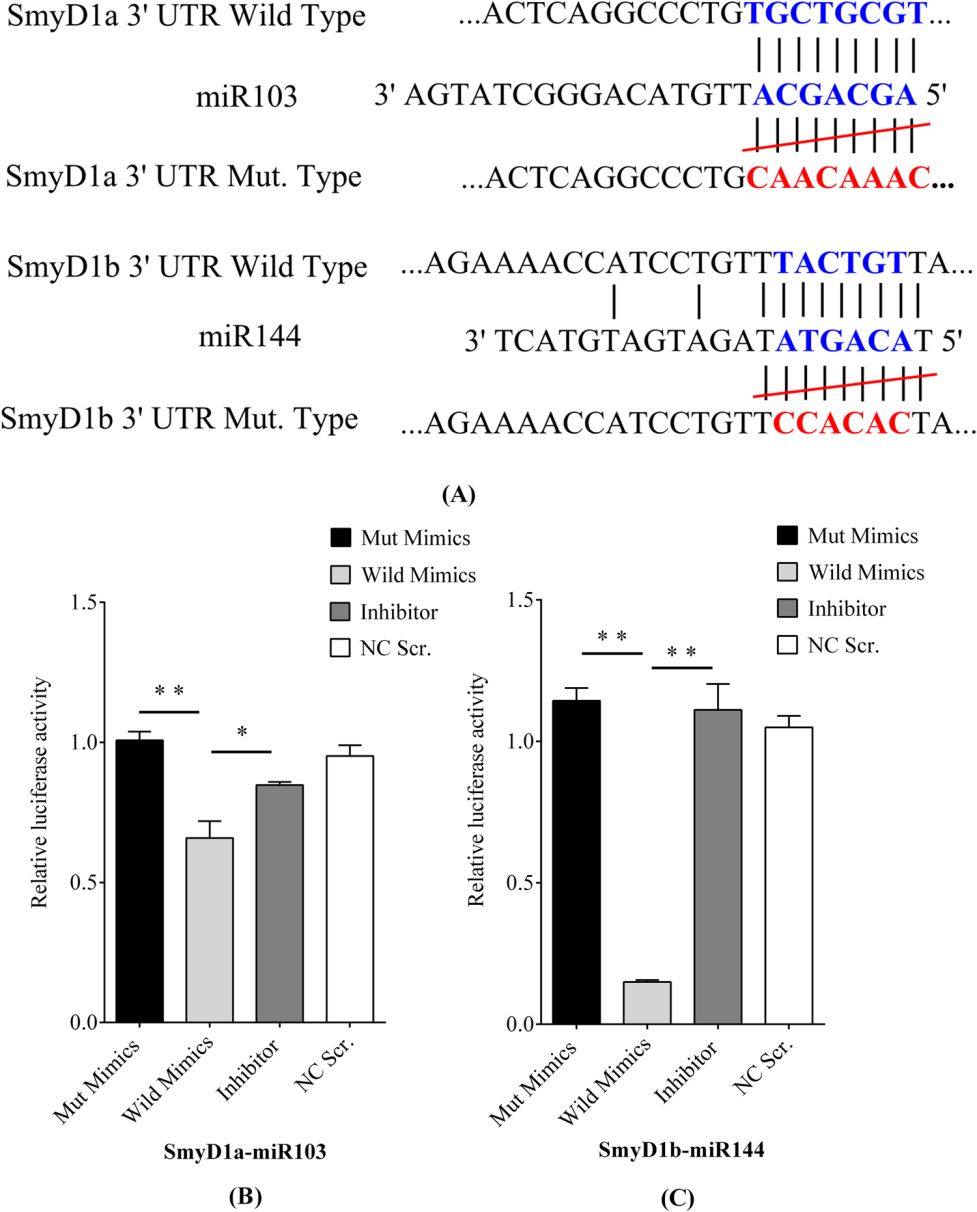
Luciferase reporter assay. (**A**) Nucleotide sequences of SmyD1a and SmyD1b wild type and mutant in the 3′-untranslated region (3′-UTR). The binding sites were marked in blue, and the mutant region marked in red. (**B**) and (**C**) The relative luciferase activities of SmyD1a and SmyD1b wild type, inhibitor and mimics NC Scr (Negative Control), the luciferase activity was normalized to *Renilla* luciferase activity.

To investigate whether SmyD1a could be directly targeted by miR-103, we engineered the luciferase reporters that have either the wild type or the mutant 3′-UTR sequence of the SmyD1a gene. The luciferase reporters were cotransfected with the miR-103 mimic into 293 T cells. As demonstrated in Fig. 3, the relative luciferase activity of SymD1a 3′-UTR wild type was significantly reduced (*p* < 0.01) when the miR-103 mimic was co-transfected with the plasmids containing the miR-103 targeting site into 293T cells, suggesting that miR-103 directly targets the SmyD1a. Similarly, to determine whether the SmyD1b gene could be directly targeted by the miR-144, the wild type and the mutant 3′-UTR sequence of the SmyD1b gene were separately constructed into the luciferase reporters. And co-transfection of the miR-144 mimic with the SmyD1b luciferase construct significantly repressed the luciferase activity (Fig. 3C). Mutation of target sites in SmyD1b completely relieved the repression.

### The putative myomiR-SmyD1 network in regulating the performance of different muscle fiber types

**Figure 4:**
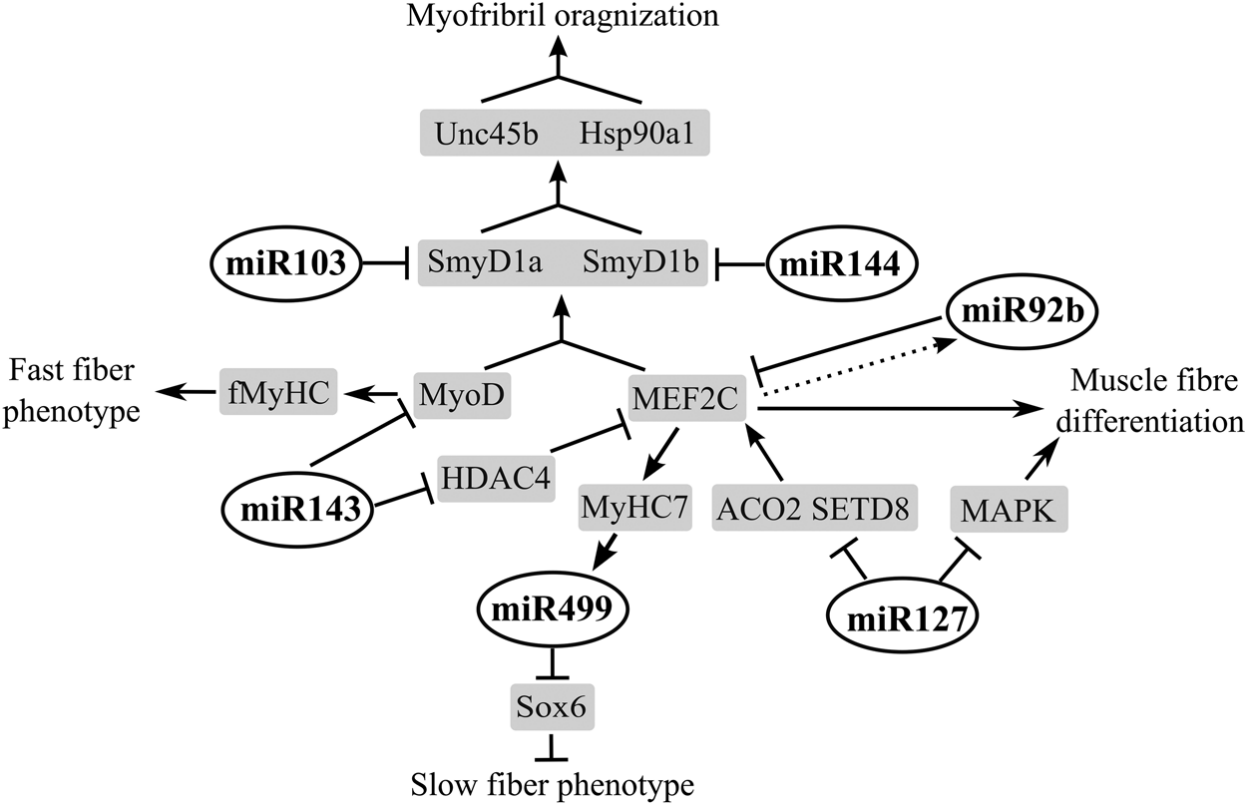
The putative myomiR-SmyD1 network in regulating the performance of different muscle fiber types. ‘↑’ means activation and ‘⊥’ means repression.

It is well known that muscle maintenance and function require transcriptional regulators, structural proteins, molecular chaperones and microRNAs. Based on published findings and our obtained data, we constructed a putative regulatory network showing the interactions of SmyD1 with other regulatory and structural proteins as well as microRNAs that are critical fast and slow muscle fiber formation and function. This network includes 6 specific miRNAs (miR-103, miR-143, miR-144, miR-499, miR-127 and miR-92b) and 11 regulatory and structural proteins (MyoD, MEF2C, HDAC4, Sox6, SETD8, MAPK, ACO2, Hsp90a1, Unc45b, fMyHC, MyHC7) (Fig. 4).

## Discussion

Fish skeletal muscles are composed of two distinct layers of tissues termed as white muscle and slow muscle. Several lines of evidence indicated that the performance of fast and slow muscles are regulated by fiber-type specific genes, or multiple proteins during fish ontogeny^23^. The isobaric tag for relative and absolute quantitation (iTRAQ) method is a sensitive and accurate for protein quantification. Several earlier reports have successfully identified protein profiles of higher vertebrates with this method. Such as in landrace and miniature pig, a total of 4431 proteins from 17,214 peptides were identified^24^. In mouse muscles, a total of 4585 peptides corresponding to 236 proteins (protein probability > 0.9) were identified and quantified^25^. In the study, we first applied the iTRAQ method to analyze the proteomic profiles of the Chinese perch skeletal muscle. The obtained data revealed that 9711 peptides and 2102 proteins were detected in Chinese perch muscles. The results indicate that iTRAQ method is applicable for fish muscle proteomic analysis.

Several muscle regulated genes and miRNAs were identified involving in muscle development and performances. MEF2C is a member of the myogenic enhancer transcription factor 2 (MEF2) family, which plays central roles in muscle fiber phenotype regulation^26^. Histone Deacetylase 4 (HDAC4) is predominantly localized to the nuclei in fast fibers in contrast to the sarcoplasm in slow fibers^27^. HDAC4 can establish the fiber type-specific transcriptional programs by repressing MEF2 activity^28^. It has been shown that overexpression of miR-143 could induce the increase of slow fibers through the HDAC4/MEF2C/MyHC7 (the myosin heavy chain in slow muscle) pathway in swine^29^. MiR-499 and MiR-208 have been shown to regulate the slow-twitch phenotype through the transcription factors (Sox6, Purβ and Sp3)/β-MyHC pathway in which Sox6 plays a vital role^9,10^. MiR-127 has been shown to target lysine methyltransferase 8 (SETD8), mitogen-activated protein kinase 4 (MAPK4) and aconitase 2 (ACO2) to regulate muscle fiber types^30–32^. In addition, there is a negative feedback circuit between miR-92b and MEF2. MEF2 can activate the expression of miR-92b, which then down-regulates MEF2 through binding to its 3′-UTR^33^.

In the study, 499 proteins were differentially expressed in two types of muscle tissues, we listed 24 proteins which participate in controlling the performance of the muscle fibers (Table 1). Among them, 11 proteins were up-regulated and 13 proteins were down-regulated comparing the fast muscles with slow muscles. Most of the differentially expressed genes or proteins are muscle structural proteins, such as myosin heavy chain (MHC), mysosin light chain (MLC), as well as muscle regulating proteins, as SmyD1a, SmyD1b, and Kelch repeat and BTB domain-containing protein 13, which may involve in controlling the performance of the two muscle fiber types. Therefore, identification of related genes or proteins could provide valuable information for regulation on skeletal muscle phenotypes.

MicroRNAs (miRNAs) are a class of evolutionally conserved non-coding RNAs of 18–25 nucleotides (nt) and they may play important gene-regulatory roles in animals and plants by pairing to the mRNAs of protein-coding genes to direct their posttranscriptional repression^34^. In the study, miRNA microarray assay revealed that 199 overlapping miRNAs were identified in two types of muscle fibers in Chinese perch. Among them, 59 miRNAs were differentially expressed between the two types of muscles. As showed in Fig. 1, 19 miRNAs exhibited an apparent expression difference between the fast and slow muscles, indicating their potential roles in controlling the performance of different muscle fiber types. Similar to our reports, in common carp, miR133a-3p and miR206 were identified in the process of skeletal muscle development in carp skeletal muscle^35^. Let7j, miR460, miR133 and miR30b have been identified and quantified with the differential expression levels in the skeletal muscle of Nile tilapia (*Oreochromis niloticus*)^36^. Those differential miRNAs may play an important role in the development and improvement of skeletal muscle. Many microRNAs, like miR-1 and miR-133, are muscle specific and they are directly involved in regulating muscle development^37^. It has been shown that miR-1 and miR-133 have distinct roles in controlling skeletal muscle proliferation and differentiation in cultured myoblasts *in vitro* and in Xenopus laevis embryos *in vivo* ^38^. miR-103 was reported to be a muscle specific miRNA that regulates myogenesis and development in higher vertebrates, such as in Cashmere Goat Skeletal Muscle^39^, while miR-144 clusted with the miR-451 was predicted to target AMPK pathway components to effect contractile differentiation of smooth muscle cells^40^. In our study, the expression levels of the miR-103 and miR-144 were comparatively assayed with Quantitative real-time PCR and the obtained data revealed that the two miRNAs were differentially expressed between the fast and slow muscles in the Chinese perch, indicating their different roles in regulating muscle fiber development or performance.

SmyD1, a member of the SmyD family, is a SET and MYND domain-containing protein that is specifically expressed in skeletal and cardiac muscles^41^. Knockout of mouse SmyD1 gene resulted in early embryonic lethality due to defective cardiac morphogenesis^18^. Recent studies have shown that zebrafish has two SmyD1 genes, SmyD1a and SmyD1b. They are important regulators in myofibril organization during myofiber maturation in zebrafish embryo^21^. Further study demonstrated that SmyD1 was a direct downstream target gene of MyoG, MyoD, SRF and MEF2C in skeletal muscle^42^. These transcriptional factors bind directly to the SmyD1 promoter region and synergistically activate its expression in C2C12 cells^43^. Further studies showed that SmyD1b might work together with two myosin chaperones Hsp90a1 and Unc45b to control sarcomere assembly during vertebrate development^44^. The Smyd1b gene was cloned from Chinese perch^20^. The Smyd1b encodes two alternatively spliced mRNAs, with the longer isoform contains an extra exon 5 encoding 13-aa insertion in the SET domain. The two transcripts showed significant higher levels of expression in skeletal muscles and heart tissues in adult Chinese perches. In the present study, we found the SmyD1a and SmyD1b proteins were differentially expressed in slow and fast muscle fibers by iTRAQ-based quantitative proteomic approach. miRNA microarray analysis showed that the miR-103 and miR-144 expression was negatively correlated with SmyD1a and SmyD1b expression in fast and slow muscles of Chinese perch, respectively. The luciferase reporter assay further verified the direct interaction between the miR-103 and SmyD1a. Furthermore, miR-144 was demonstrated specifically to target the 3′-untranslated region of the SmyD1b gene (Fig. 3), the gene structure similar to that as reported in zebrafish^45^. Therefore, the sch-miR-103 and miR-144 could act to control the performance of the different muscle fiber types by targeting the SmyD1a and SmyD1b genes, respectively. Moreover, the constructed miRNA-SmyD1 interaction network revealed that the miRNAs participated in controlling the performance of different muscle fiber types of Chinese perch through multiple transcriptional pathways.

## Methods and Materials

### Ethics statement

The fish were monitored daily during the entire experimental period. They were monitored for swimming behavior and eating activity. All fish used in the study were healthy. No animals became severely ill or died at any time prior to the experimental endpoint. We had an IACUC approved by the Institutional Animal Care and Use Committee (IACUC) of Changsha University (permit #20128945-1). All surgeries were performed under sodium pentobarbital or tricaine methanesulfonate (MS-222) anesthesia, and every effort was made to minimize the animal suffering.

All experiments were conducted at the Changsha University, and all experimental procedures and methods were performed in accordance with relevant guidelines and regulations by the Committee (IACUC).

### Sample collection and preparation

Chinese perch (*Siniperca chuasti*) of two years old were obtained from Hunan Fisheries Science Institute, Changsha, Hunan, China. After the fish were dissected, red muscle and white muscle were immediately preserved in liquid nitrogen, and then stored at −80 °C for further processing.

### Protein preparation and iTRAQ Labeling

Total proteins were extracted from fish muscles according to the procedure previously described by Zhang *et al*.^46^. Protein identification and quantification were performed using iTRAQ as described by Zhang *et al*.^47^. 100 μg of protein from each sample was digested with Trypsin Gold (Promega, WI, USA) at the ratio of protein:trypsin (20:1) at 37 °C for 4 hours. The sample was digested again using Trypsin Gold with the ratio of protein:trypsin = 20:1 one more time and digest for a total of 8 hours. After trypsin digestion, the samples were dried by vacuum centrifugation. The protein samples were redissolved in 0.5 M TEAB. The iTRAQ labeling was performed using iTRAQ Reagent 6-plex Kit (AB SCIEX) according to the manufacturer’s protocol (Supplementary Figure S2). The protein samples were labeled as 113 (SM1), 114 (SM2), 115 (SM3), 116 (FM1), 117 (FM2), and 118 (FM3). SM means slow muscle and FM means fast muscle. Slow muscle (I_113_, I_114_, I_115_) and fast msucle (I_116_, I_117_, I_118_) were compared using technical replicates, i.e., three channels per sample class. The peptides labeled with respective isobaric tags, incubated for 2 h. The iTRAQ labeled peptides were fractionated using SCX.

### SCX Chromatography

The SCX chromatography was characterized using the ShimadzuLC-20AB HPLC Pump system. Peptides from trypsin digestion was reconstituted with 4 mL buffer A (25 mM NaH_2_PO_4_ in 25% ACN, pH2.7) and loaded onto a 4.6 × 250 mm Ultremex SCX column containing 5-μm particles (Phenomenex). The peptides were eluted at a flow rate of 1 mL/min with a gradient of buffer A for 10 min, 5–35% buffer B (25 mM NaH_2_PO_4_, 1 M KCl in 25% ACN, pH2.7) for 11 min, and 35–80% buffer B for 1 min. The system was then maintained in 80% buffer B for 3 min before equilibrating with buffer A for 10 min prior to the next sample injection. Elution was monitored by measuring absorbance at 214 nm, and fractions were collected every 1 min. The eluted peptides were pooled as 20 fractions, desalted by StrataXC18 column (Phenomenex) and vacuum-dried.

### LC-ESI-MSMS analysis

LC-ESI-MSMS analysis was carried out based on Triple TOF 5600. Each fraction was resuspended in buffer A (5% ACN, 0.1% FA) and centrifuged at 20000 g for 10 min, the final concentration of peptide was about 0.5.g/.l on average. 10.l supernatant was loaded on a LC-20AD nanoHPLC (Shimadzu, Kyoto, Japan) by the autosampler onto a 2 cm C18 trap column. The peptides were eluted onto a 10 cm analytical C18 column (inner diameter 75) packed in-house. The samples were loaded at 8.L/min for 4 min, then the 41 min gradient was run at 300 nL/min starting from 5%B (95% ACN, 0.1% FA) to 35%B, followed by a 5 min linear gradient to 80%, and maintenance at 80%B for 5 min, and finally return to 5% in 1 min.

Data acquisition was performed with a TripleTOF 5600 system (AB SCIEX, Concord, ON) fitted with a Nanospray III source (AB SCIEX, Concord, ON) and a pulled quartz tip as the emitter (New objectives, Woburn, MA). Data was acquired using an ion spray at a voltage of 2.5 kV, curtaining gas of 30 psi, nebulizer gas of 15 psi, and an interface heater temperature of 150 °C. The MS was operated with a RP of greater than or equal to 30, 000 FWHM for TOF MS scans. For IDA, survey scans were acquired in 250 ms and as many as 30 production scans were collected if exceeding a threshold of 120 counts per second (counts/s) and with a 2+ to 5+ charge-state. Total cycle time was fixed to 3.3 s. Q2 transmission window was 100 Da for 100%. Four time bins were summed for each scan at a pulser frequency value of 11 kHz through monitoring of the 40 GHz multichannel TDC detector with four-anode channel detect ion. A sweeping collision energy setting of 35 ± 5 eV coupled with iTRAQ adjusts rolling collision energy was applied to all precursor ions for collision-induced dissociation. Dynamic exclusion was set for 1/2 of peak width (15 s), and then the precursor was refreshed off the exclusion list.

### Protein identification and quantification

The Mascot^22^ protein identification software was used. The raw MS/MS data was converted into MGF format by ProteoWizardtool msConvert, and the exported MGF files were searched using Mascot version 2.3.02 in this project against the selected data base: I-mXrJX003 (42173 sequences, downloaded on the 2015-10-21). The search parameters included the following: peptide mass tolerance of 0.05 Da, fragment mass tolerance of 0.1 Da, sample type of iTRAQ 6 plex (peptide-labeled), cysteine alkylation of iodoacetamide, and digestion of trypsin. The peak intensity of the exosome peptide segmenteport was analyzed by Proteome Discoverer 1.4 software. The false discovery rate (FDR) of protein identification was set less than 0.01. At least one unique peptide was required for each identified protein.

An automated software called IQuant for quantitatively analyzing the labeled peptides with isobaric tags. It integrates Mascot Percolator and advanced statistical algorithms to process the MS/MS signals generated from the peptides labeled by isobaric tags. The mean of all labeled samples was used as a reference for calculating the iTRAQ ratios of all reporter ions. The defined quantification ratios for the protein group were calculated as the median of all PSMs belonging to the protein group. The final ratio obtained from the relative protein quantification was normalized to the median average protein quantification ratio. The fold change in protein abundance between the two compared experimental groups was calculated on the basis of the average value of three replicates from the fast muscle and slow muscle groups. Finally, differentially proteins were defined by its quantitative signals with Q-values ≤ 0.05 and Fold change ≥ 1.2 or ≤0.83. And all differentially expressed proteins were defined in at least 2 replicated experiments^48^.

All proteomics data have been deposited into PeptideAtlas (ftp://PASS00994:PA3955to@ftp.peptideatlas.org/).

### Bioinformatics analysis

Functional analysis was conducted using gene ontology (GO) annotations by Blast2GO software (v4.5 pipeline 5), and the proteins were categorized according to their biological processes, molecular functions, and cellular localizations. The differentially expressed proteins were further assigned to the Kyoto Encyclopedia of genes and genomes (KEGG) database (http://www.genome.jp/kegg/pathway.html).

### miRNA Microarray Analysis

The miRNA microarrays were used to analyze miRNA expression pattern in fast and slow muscles (LC-Bio Hangzhou, China) as referenced by Xu *et al*.^49^. Chip hybridizations were performed overnight on a μParaflo microfluidic chip using a microcirculation pump (Atactic Technologies). After hybridization, signals were detected using tag-specific Cy3 and Cy5 dyes. Hybridization images were collected using a laser scanner (GenePix 4000B, Molecular Devices, Sunnyvale, CA, USA) and digitized by Array-Pro image analysis software (Media Cybernetics, Bethesda, MD, USA). Finally, hybridization signals were detected and quantified, and data were analyzed by first subtracting the background and then normalizing the signals with a cyclic LOWESS filter (Locally-weighted Regression). All microarray data was deposited in GEO DataSets (accession number GSE97173).

### Quantitative real-time PCR

Total RNA was extracted using Trizol reagent (Invitrogen) and the miRNAs were extracted with the miRNeasy kit (Qiagen). The expression of miRNA was determined using the one step PrimeScript miRNA cDNA synthesis kit (TaKaRa, Dalian, China) using Rpl13 as an endogenous control. All the primers used in the real time PCR are listed in Table 3. The relative amount of miRNA and mRNAs were calculated using the 2^−ΔΔct^ method, and all quantitative data presented were the mean ± SEM.

**Table 3:** Primers used for miRNA quantitative real-time PCR (qPCR).

Name	Sequence (5′−3′)
miR-103-F	AGCAGCATTGTACAGGGCTATGA
miR-144-F	TACAGTATAGATGATGTACTAT
RPL-13-F	CACAAGAAGGAGAAGGCTCGGGT
RPL-13-R	TTTGGCTCTCTTGGCACGGAT

The forward miRNA primers used for detection were above; the reverse miRNA primers used for detection was the universal downstream primer (Uni-miR qPCR Primer, 10 μmol/L, Takara).

### Prediction of the target of miRNAs

In order to predict the target genes of miR-103 and miR-144, the TargetScan Fish 6.2 (http://www.targetscan.org/fish_62/) was used for target prediction. The seed site and the target gene were also matched by close manual examination.

### 3′-UTR luciferase reporter assay

The procedure for the 3′-UTR luciferase reporter assay was done as described by Stockley *et al*.^50^. Briefly, the 3′-UTR luciferase wild type reporter plasmids were constructed by introducing gene 3′-UTR carrying putative miRNA binding sites into the downstream of the firefly luciferase gene. Meanwhile, target site of eight base pairs of the putative miRNA binding sites were replaced to generate mutant type. Luciferase assays were carried out in 293T cells. Cells were transfected with either wild-type or mutant constructs, with mimics or mimics and inhibitors or the negative control mimic. And 48 hours later, Dual-Glo™ Luciferase Assay System (E2920, Promega) was performed to detect the luminescence.

### Statistical Analysis

Statistical significance was analyzed using the Student’s t-test (paired or unpaired) or one-way analysis of variance (ANOVA) with SPSS statistical software 14.0 (SPSS Inc., USA). Data were presented as mean ± standard deviation (SD). Differences were considered to be statistically significant at *p* < 0.05.

## Electronic supplementary material

Supplementary information

Dataset 1

Dataset 2

Dataset 3

Dataset 4
