# Supplementary material for: Proteomic and microRNA Transcriptome Analysis revealed the microRNA-SmyD1 network regulation in Skeletal Muscle Fibers performance of Chinese perch
Source: Sci Rep. 2017 Nov 28;7:16498. doi: 10.1038/s41598-017-16718-2 (PMC5705591; doi:10.1038/s41598-017-16718-2)
Supplement: Supplementary file 1 — Supplementary information [file 41598_2017_16718_MOESM1_ESM.pdf]

Supplementary informations

Proteomic and microRNA Transcriptome Analysis revealed the microRNA-SmyD1 network regulation in Skeletal Muscle Fibers performance of Chinese perch

WuYing Chu[1,2#], FangLiang Zhang[1,2#], Rui Song[3],YuLong Li[1,2], Ping Wu[1,2], Lin Chen[1,2], Jia Cheng[1,2] , ShaoJun Du[1,4] & JianShe Zhang[1,2*]

1. Department of Bioengineering and Environmental Science, Changsha University, Changsha, Hunan 410003, China;

2. Collaborative Innovation Center for Efficient and Health Production of Fisheries in Hunan Province, Changde 415000, China;

3. Institute of Hunan Aquaculture and Fisheries, Changsha, 410005, China

4. Institute of Marine and Environmental technology, Department of Biochemistry and Molecular Biology, University of Maryland, USA.

Supplementary Table S1: All differentially expressed proteins in Fast and Slow muscle.

Supplementary Table S2. KEGG pathway analysis of all differential proteins in Fast and Slow muscle.

Nunber	Pathway	Differential Proteins with pathway annotation	*P*-value	Pathway ID
1	Metabolic pathways	143(31.78%)	7.36E-25	ko05410
2	Dilated cardiomyopathy	114(25.33%)	5.11E-24	ko05414
3	Hypertrophic cardiomyopathy (HCM)	113(25.11%)	3.35E-17	ko00190
4	Cardiac muscle contraction	68(15.11%)	9.68E-17	ko05012
5	Alzheimer's disease	65(14.44%)	2.38E-16	ko05010
6	Huntington's disease	64(14.22%)	8.48E-15	ko05016
7	Parkinson's disease	61(13.56%)	1.25E-14	ko04260
8	Oxidative phosphorylation	56(12.44%)	1.19E-12	ko01100

9	Regulation of actin cytoskeleton	50(11.11%)	3.24E-07	ko04270
10	Tight junction	46(10.22%)	2.80E-06	ko00630
11	Vascular smooth muscle contraction	41(9.11%)	4.76E-06	ko05416
12	Salmonella infection	41(9.11%)	4.98E-06	ko00020
13	Viral myocarditis	39(8.67%)	7.14E-06	ko05132
14	Focal adhesion	28(6.22%)	1.78E-05	ko04530
15	Calcium signaling pathway	26(5.78%)	5.91E-05	ko04810
16	Valine, leucine and isoleucine degradation	23(5.11%)	5.91E-05	ko00280
17	Glycolysis / Gluconeogenesis	22(4.89%)	0.000281522	ko00650
18	Pyruvate metabolism	18(4%)	0.000502158	ko00620
19	Citrate cycle (TCA cycle)	17(3.78%)	0.007591757	ko00010
20	Fatty acid metabolism	15(3.33%)	0.009097855	ko00260
21	Arrhythmogenic right ventricular cardiomyopathy (ARVC)	15(3.33%)	0.009460833	ko00071
22	Glyoxylate and dicarboxylate metabolism	13(2.89%)	0.01759566	ko04020
23	Leukocyte transendothelial migration	13(2.89%)	0.01799842	ko04964
24	Propanoate metabolism	12(2.67%)	0.04148438	ko04510
25	Amoebiasis	12(2.67%)	0.04415254	ko00640
26	Arginine and proline metabolism	12(2.67%)	0.04645585	ko04146
27	Influenza A	12(2.67%)	0.04846541	ko00290
28	Peroxisome	11(2.44%)	0.05021915	ko05143
29	Butanoate metabolism	10(2.22%)	0.05038605	ko00310
30	Pancreatic secretion	10(2.22%)	0.05159202	ko00380
31	Endocytosis	10(2.22%)	0.05654465	ko05146
32	MAPK signaling pathway	10(2.22%)	0.06049843	ko00400
33	Pathogenic Escherichia coli infection	10(2.22%)	0.0731831	ko00062
34	Pathways in cancer	10(2.22%)	0.08780752	ko00330
35	Lysine degradation	9(2%)	0.09923084	ko00830
36	Protein processing in endoplasmic reticulum	9(2%)	0.1362931	ko05412
37	Glycine, serine and threonine metabolism	8(1.78%)	0.1491243	ko00250
38	Tryptophan metabolism	8(1.78%)	0.1497134	ko04974
39	Protein digestion and absorption	8(1.78%)	0.1523855	ko04972
40	Starch and sucrose metabolism	8(1.78%)	0.1868277	ko04670
41	Toxoplasmosis	8(1.78%)	0.1957925	ko00052

42	ECM-receptor interaction	8(1.78%)	0.201204	ko05020
43	Tuberculosis	8(1.78%)	0.2188971	ko00500
44	HTLV-I infection	8(1.78%)	0.2353864	ko04070
45	Purine metabolism	8(1.78%)	0.2353864	ko00040
46	Prion diseases	7(1.56%)	0.2353864	ko00350
47	PPAR signaling pathway	7(1.56%)	0.2461707	ko00471
48	Gastric acid secretion	7(1.56%)	0.2461707	ko00300
49	Phagosome	7(1.56%)	0.2975185	ko05145
50	Epstein-Barr virus infection	7(1.56%)	0.2975185	ko04512
51	Ribosome	7(1.56%)	0.3117522	ko04350
52	Proximal tubule bicarbonate reclamation	6(1.33%)	0.3172724	ko00410
53	African trypanosomiasis	6(1.33%)	0.3172724	ko05150
54	Beta-Alanine metabolism	6(1.33%)	0.3284332	ko05164
55	Staphylococcus aureus infection	6(1.33%)	0.3557501	ko05222
56	Legionellosis	6(1.33%)	0.3591015	ko05144
57	Salivary secretion	6(1.33%)	0.3591015	ko04975
58	Adherens junction	6(1.33%)	0.3591015	ko00360
59	Bacterial invasion of epithelial cells	6(1.33%)	0.3591015	ko00072
60	Insulin signaling pathway	6(1.33%)	0.3670159	ko05134
61	Fatty acid elongation	5(1.11%)	0.3751965	ko03320
62	Alanine, aspartate and glutamate metabolism	5(1.11%)	0.3885656	ko00340
63	Galactose metabolism	5(1.11%)	0.3885656	ko04930
64	Small cell lung cancer	5(1.11%)	0.4113912	ko05034
65	Alcoholism	5(1.11%)	0.417053	ko04970
66	Fructose and mannose metabolism	5(1.11%)	0.4318429	ko00592
67	Pentose phosphate pathway	5(1.11%)	0.4318429	ko00130
68	Measles	5(1.11%)	0.4318429	ko03020
69	Dopaminergic synapse	5(1.11%)	0.4318429	ko00460
70	Systemic lupus erythematosus	5(1.11%)	0.4571054	ko04744
71	Shigellosis	5(1.11%)	0.4653309	ko00270
72	Antigen processing and presentation	5(1.11%)	0.5252932	ko03018
73	Oocyte meiosis	5(1.11%)	0.5332757	ko05133
74	Cysteine and methionine metabolism	4(0.89%)	0.5332757	ko04973
75	RNA degradation	4(0.89%)	0.545793	ko01040
76	Complement and coagulation cascades	4(0.89%)	0.545793	ko00053

77	Phototransduction - fly	4(0.89%)	0.545793	ko00670
78	Amyotrophic lateral sclerosis (ALS)	4(0.89%)	0.5703125	ko00051
79	Amphetamine addiction	4(0.89%)	0.5718597	ko00120
80	Vibrio cholerae infection	4(0.89%)	0.5718597	ko00900
81	Cell adhesion molecules (CAMs)	4(0.89%)	0.5718597	ko04614
82	Long-term potentiation	4(0.89%)	0.5818076	ko04610
83	Gap junction	4(0.89%)	0.5818076	ko04745
84	Neurotrophin signaling pathway	4(0.89%)	0.5979858	ko04730
85	Valine, leucine and isoleucine biosynthesis	3(0.67%)	0.5979858	ko00561
86	Retinol metabolism	3(0.67%)	0.5979858	ko05214
87	Phosphatidylinositol signaling system	3(0.67%)	0.6182061	ko00030
88	Pentose and glucuronate interconversions	3(0.67%)	0.6182061	ko05162
89	Tyrosine metabolism	3(0.67%)	0.6238065	ko04966
90	TGF-beta signaling pathway	3(0.67%)	0.6238065	ko04960
91	Histidine metabolism	3(0.67%)	0.6342434	ko05014
92	Type II diabetes mellitus	3(0.67%)	0.6565242	ko04962
93	Pertussis	3(0.67%)	0.6565242	ko00760
94	Carbohydrate digestion and absorption	3(0.67%)	0.6627991	ko04728
95	Long-term depression	3(0.67%)	0.6729704	ko04144
96	Glycerolipid metabolism	3(0.67%)	0.6774286	ko00534
97	Glioma	3(0.67%)	0.6774286	ko04940
98	Vasopressin-regulated water reabsorption	3(0.67%)	0.6774286	ko00524
99	Nicotinate and nicotinamide metabolism	3(0.67%)	0.6822348	ko05031
100	Amino sugar and nucleotide sugar metabolism	3(0.67%)	0.6910183	ko05340
101	Pyrimidine metabolism	3(0.67%)	0.6910183	ko04115
102	Bile secretion	3(0.67%)	0.6910183	ko00982
103	Melanogenesis	3(0.67%)	0.702606	ko04010
104	Apoptosis	3(0.67%)	0.7072782	ko04971
105	GnRH signaling pathway	3(0.67%)	0.7086678	ko00520
106	Glutathione metabolism	3(0.67%)	0.7086678	ko00240
107	Wnt signaling pathway	3(0.67%)	0.7256338	ko05110
108	Herpes simplex infection	3(0.67%)	0.7256338	ko04514
109	Spliceosome	3(0.67%)	0.7480087	ko04978

110	Fc gamma R-mediated phagocytosis	3(0.67%)	0.7480087	ko04664
111	Proteasome	3(0.67%)	0.7480087	ko05210
112	Phenylalanine, tyrosine and tryptophan biosynthesis	2(0.44%)	0.7480087	ko04740
113	Malaria	2(0.44%)	0.7545754	ko05152
114	Fat digestion and absorption	2(0.44%)	0.7566333	ko05130
115	Phenylalanine metabolism	2(0.44%)	0.7570103	ko05223
116	Synthesis and degradation of ketone bodies	2(0.44%)	0.7570103	ko05216
117	Phototransduction	2(0.44%)	0.7570103	ko05310
118	Biosynthesis of unsaturated fatty acids	2(0.44%)	0.7751999	ko05322
119	Ascorbate and aldarate metabolism	2(0.44%)	0.7751999	ko05131
120	One carbon pool by folate	2(0.44%)	0.7751999	ko04612
121	Collecting duct acid secretion	2(0.44%)	0.7814227	ko05166
122	Aldosterone-regulated sodium reabsorption	2(0.44%)	0.7943662	ko04976
123	Primary immunodeficiency	2(0.44%)	0.7957272	ko05140
124	p53 signaling pathway	2(0.44%)	0.7988778	ko04720
125	Drug metabolism - cytochrome P450	2(0.44%)	0.8169913	ko05320
126	Mineral absorption	2(0.44%)	0.8169913	ko05330
127	Fc epsilon RI signaling pathway	2(0.44%)	0.8169913	ko04672
128	Colorectal cancer	2(0.44%)	0.8286782	ko04916
129	Olfactory transduction	2(0.44%)	0.8291175	ko04540
130	Leishmaniasis	2(0.44%)	0.8374921	ko04520
131	Metabolism of xenobiotics by cytochrome P450	2(0.44%)	0.8677757	ko00980
132	NF-kappa B signaling pathway	2(0.44%)	0.8942937	ko04064
133	Transcriptional misregulation in cancer	2(0.44%)	0.8956447	ko05100
134	mRNA surveillance pathway	2(0.44%)	0.896246	ko00860
135	Natural killer cell mediated cytotoxicity	2(0.44%)	0.896246	ko05032
136	Lysosome	2(0.44%)	0.896246	ko04640
137	B cell receptor signaling pathway	2(0.44%)	0.896246	ko04726
138	Endocrine and other factor-regulated calcium reabsorption	2(0.44%)	0.9036585	ko04210
139	Ubiquitin mediated proteolysis	2(0.44%)	0.9108422	ko04145
140	Chemokine signaling pathway	2(0.44%)	0.9157986	ko05202
141	D-Glutamine and D-glutamate metabolism	1(0.22%)	0.9157986	ko03015

142	Lysine biosynthesis	1(0.22%)	0.921122	ko04912
143	Alpha-Linolenic acid metabolism	1(0.22%)	0.9260466	ko05200
144	Ubiquinone and other terpenoid-quinone biosynthesis	1(0.22%)	0.9339648	ko00230
145	RNA polymerase	1(0.22%)	0.9412206	ko05211
146	Cyanoamino acid metabolism	1(0.22%)	0.9412206	ko05142
147	Primary bile acid biosynthesis	1(0.22%)	0.9412206	ko05323
148	Terpenoid backbone biosynthesis	1(0.22%)	0.9412206	ko04723
149	Renin-angiotensin system	1(0.22%)	0.9470751	ko04650
150	Glycosaminoglycan biosynthesis - heparan sulfate	1(0.22%)	0.9470751	ko04142
151	Type I diabetes mellitus	1(0.22%)	0.9473227	ko04114
152	Butirosin and neomycin biosynthesis	1(0.22%)	0.95577	ko04727
153	Non-small cell lung cancer	1(0.22%)	0.9598757	ko04722
154	Thyroid cancer	1(0.22%)	0.9657128	ko00480
155	Asthma	1(0.22%)	0.966724	ko04380
156	Autoimmune thyroid disease	1(0.22%)	0.9670883	ko04662
157	Allograft rejection	1(0.22%)	0.9811756	ko05160
158	Intestinal immune network for IgA production	1(0.22%)	0.9811756	ko04012
159	Porphyrin and chlorophyll metabolism	1(0.22%)	0.9811756	ko04370
160	Morphine addiction	1(0.22%)	0.9811756	ko04724
161	Hematopoietic cell lineage	1(0.22%)	0.982345	ko05169
162	Serotonergic synapse	1(0.22%)	0.9841232	ko04961
163	Renal cell carcinoma	1(0.22%)	0.985729	ko04310
164	Chagas disease (American trypanosomiasis)	1(0.22%)	0.985807	ko04141
165	Rheumatoid arthritis	1(0.22%)	0.9858454	ko04725
166	Retrograde endocannabinoid signaling	1(0.22%)	0.9858454	ko05215
167	GABAergic synapse	1(0.22%)	0.9888562	ko04910
168	Osteoclast differentiation	1(0.22%)	0.9909143	ko05168
169	Hepatitis C	1(0.22%)	0.992463	ko04120
170	ErbB signaling pathway	1(0.22%)	0.9927715	ko03040
171	VEGF signaling pathway	1(0.22%)	0.9927715	ko04666
172	Glutamatergic synapse	1(0.22%)	0.9939889	ko04110
173	Cholinergic synapse	1(0.22%)	0.9954475	ko04062
174	Prostate cancer	1(0.22%)	0.995449	ko03050
175	Cell cycle	1(0.22%)	0.996607	ko04660

176	T cell receptor signaling pathway	1(0.22%)	0.998563	ko00970
177	Aminoacyl-tRNA biosynthesis	1(0.22%)	0.9991903	ko04360
178	Axon guidance	1(0.22%)	0.9996983	ko03010

Supplementary Table S3: All differential miRNAs in Fast and Slow muscle.

Supplementary Table S4: GO enrichment of all differential proteins.

Supplementary Table S5: All proteins identification and quantification.

Supplementary Figure S1. GO annotation for identified expressed proteins in fast and slow muscle. (A) Biological processes, (B) Cellular function and (C) Molecular compartment.

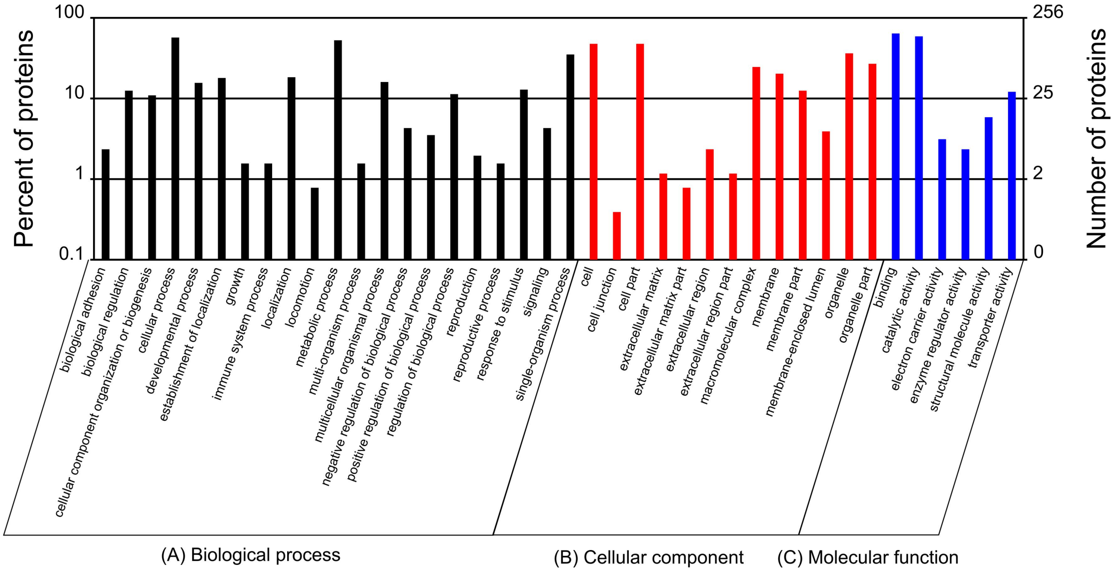

Supplementary Figure S2. Experimental Procedures of iTRAQ experiment. This figure shows the main procedures of the experiment of iTRAQ quantitative proteomics. (1) Extract protein from samples. (2) Reductive alkylation. (3) Measuring the concentration of the protein with bradford method. (4) SDS-PAGE. (5) Digest proteins in gel and elute peptides. (6) iTRAQ Labeling. (7) Mix peptides with a proportion of 1:1. (8) SCX Chromatography. (9) Liquid chromatography coupled with tandem mass spectrometry(LCMS/MS).

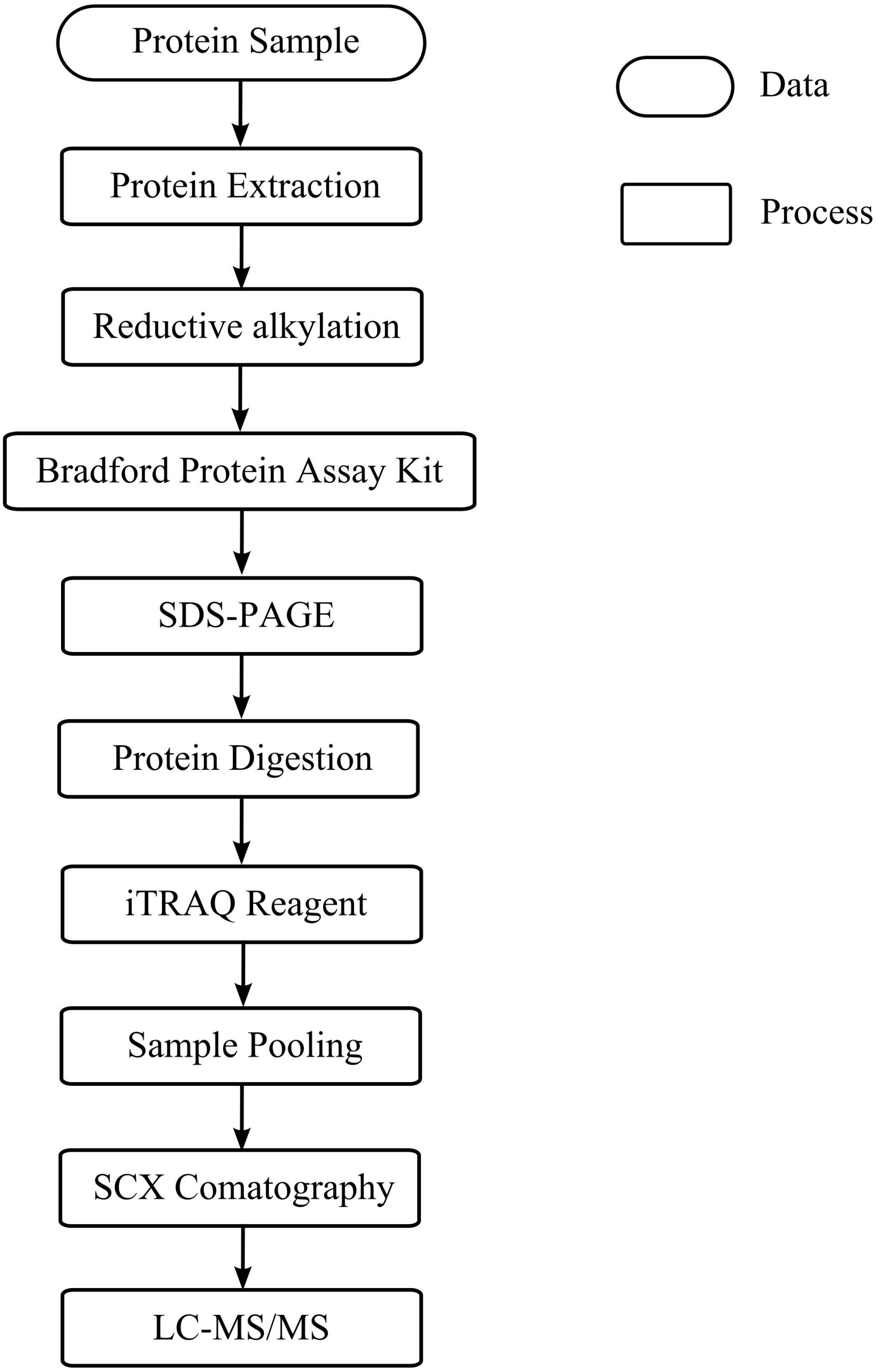

Methods

Computation of a protein ratio

The computation of a Protein Ratio A protein ratio c(p_i) is obtained by combining the ratios measured from the MS/MS spectra of its peptide spectra like

$c(s_{i,j})=\log(I_{116,i,j}/I_{113,i,j})$. The design of the test data sets, with slow muscle and fast muscle shared peptides at different ratios, was ideal to investigate the selection of the peptides integrated in the protein ratio computation. The specific peptides can be used[1,2], and even spectra from specific peptides must be filtered by eliminating outliers, most likely because of coeluting material[3]. We write S'_i as the subset of spectra for protein *i* after selection.

Several options are available to summarize the multiple spectrum ratios into a single protein ratio, and we considered usual estimators such as the median and the average (both trimmed here because of the outliers elimination) and three different weighted averages (intensity-based Multi-Q[4], or weighted by either the standard deviation $(f(x))^{1/2}$ or the variance $f(x)$. A modified boosted median evaluated on half the data consisting of the most extreme ratios was also added to address commonly observed underestimated iTRAQ ratios[3,5], which are also assumed to be caused by coeluting material. As comparison criteria, accuracy and stability (limited variance) of the estimations were considered as well as the number of spectra available influence. We found no major differences for ratios smaller than 1:10 (or 10:1), and as expected, the larger the number of available spectra, the more accurate the estimations. For larger ratios, differences in variance and accuracy become visible and the boosted median slightly outperforms the other candidates in accuracy. Nonetheless, the weighted average

$$c(p_i) = \sum_{j \in s'_i} \alpha_{i,j} c(s_{i,j}),$$

with $\alpha_{i,j} \propto (\mathrm{Var}(c(s_{i,j})))^{-1} = (f(\log(I_{113,i,j}))+f(\log(I_{116,i,j})))^{-1}$ and $\sum_j \alpha_{i,j} = 1$, has the smallest variance and is sufficiently accurate for correct biological interpretation. Based on this, we selected *i* as $c(p_i)$ estimator. The protein ratio $c(p_i)$ must be supplemented by its variance estimate to determine its reliability, so we set

$$Var(c(p_i)) = \max\{V_{estim,i}, V_{spectrum,i}\}$$

to verify it[6].

Reference

[1] Jin, S., Daly, D. S., Springer, D. L. & Miller, J. H. The effects of shared peptides on protein quantitation in label-free proteomics by LC/MS/MS. *J. Proteome Res.* **7**, 164-169 (2007).

[2] Usaite, R., Wohlschlegel, J., Venable, J. D., Park, S. K. & Yates Iii, J. R. Characterization of global yeast quantitative proteome data generated from the wild-type and glucose repression saccharomyces cerevisiae strains: the comparison of two quantitative methods. *J. Proteome Res.* **7**, 266-275 (2008).

[3] Ow, S. Y., Salim, M., Noirel, J., Evans, C. & Wright, P. C. iTRAQ underestimation in simple and complex mixtures: "the good, the bad and the ugly". *J. Proteome Res.* **8**, 5347-5355 (2009).

[4] Lin, W. T., Hung, W. N., Yian, Y. H., Wu, K. P. & Hsu, W. L. Multi-Q: a fully automated tool for multiplexed protein quantitation. *J. Proteome Res*. **5**, 2328-2338 (2006).

[5] Bantscheff, M., Boesche, M., Eberhard, D., Matthieson, T. & Kuster, B. Robust and sensitive iTRAQ quantification on an LTQ Orbitrap mass spectrometer. *Mol Cell Proteomics*. **7**, 1702-1713 (2008).

[6] Breitwieser, F. P. Müller, A., Dayon, L., Köcher, T. & Bennett, K. L. General statistical modeling of data from protein relative expression isobaric tags. *J. Proteome Res.* **10**, 2758-2766 (2011).
